# A 73,128 bp *de novo* deletion encompassing the *OPN1LW/OPN1MW* gene cluster in sporadic Blue Cone Monochromacy: a case report

**DOI:** 10.1186/s12881-018-0623-8

**Published:** 2018-06-26

**Authors:** Elena Buena-Atienza, Fadi Nasser, Susanne Kohl, Bernd Wissinger

**Affiliations:** Institute for Ophthalmic Research, Centre for Ophthalmology, University of Tuebingen, Elfriede-Aulhorn 7, D-72076 Tuebingen, Germany

**Keywords:** *De novo* mutations, Sporadic cases, Blue Cone Monochromacy, Colour vision deficiency, Alu-mediated recombination, Retinal dystrophy

## Abstract

**Background:**

Blue Cone Monochromacy (BCM) is a rare congenital cone dysfunction disorder with X-linked recessive mode of inheritance. BCM is caused by mutations at the *OPN1LW/MW* cone opsin gene cluster including deletions of the locus control region (LCR) and/or parts of the gene cluster. We aimed at investigating the clinical presentation, genetic cause and inheritance underlying a sporadic case of BCM.

**Case presentation:**

We report a 24-year-old male presenting with congenital photophobia, nystagmus and colour vision abnormalities. There was no history of retinal dystrophy in the family. Clinical diagnosis of BCM was supported by genetic investigations of the patient and his family members. Molecular genetic analysis of the *OPN1LW/OPN1MW* gene cluster revealed a novel deletion of about 73 kb in the patient encompassing the LCR. The deletion was absent in the X-chromosomes of both the mother and transmitting grandfather.

**Conclusions:**

The present report provides the clinical findings and the genetic basis underlying a sporadic BCM case which is caused by a *de novo* deletion within the *OPN1LW/MW* gene cluster originating from the mother’s germline due to Alu-repeat mediated recombination. This is the first report of a *de novo* deletion resulting in BCM, highlighting the importance to consider BCM and perform genetic testing for this condition in male patients with cone dysfunction also in the absence of a positive family history.

## Background

Blue Cone Monochromacy (BCM; OMIM#303700) is characterized by reduced visual acuity, photophobia, colour vision deficiency and it is regularly accompanied by congenital nystagmus and myopia [1]. The clinical findings in BCM patients largely overlap with those of achromatopsia patients. An X-linked recessive pattern of inheritance is a practical distinctive trait of BCM. Yet, lack of family history in simplex cases hinders prioritization of genetic tests. In addition, genetic testing for BCM is not regularly available at most diagnostic genetic testing laboratories due to the complexity of the opsin gene cluster.

The genes for the long wavelength sensitive (*OPN1LW*; LW opsin gene) and middle wavelength sensitive (*OPN1MW*; MW opsin gene) pigments in LW and MW cone photoreceptors are located on the X-chromosome and arranged as a head-to-tail tandem array in the so-called *OPN1LW/OPN1MW* gene cluster. The first position in the cluster is generally occupied by an *OPN1LW* gene copy followed by one or multiple *OPN11MW* gene copies. Each gene copy possesses a direct upstream promoter, while the expression of the genes in the gene cluster is regulated by an upstream locus control region (LCR). The LCR is a 0.6 kb *cis*-regulatory sequence essential to drive expression in a distance dependent manner so that essentially only the first two gene copies within the gene cluster are expressed [2].

Deletions affecting the *OPN1LW/OPN1MW* gene cluster on Xq28 have been estimated to account for 30–40% of all mutations found in BCM patients [3, 4]. Whereas deletions may be restricted to the LCR region, a number of deletions have been reported to extend towards the human cone opsin gene cluster, i.e. deleting partially or completely the *OPN1LW* and/or *OPN1MW* gene copies [1, 3–7].

In this report, we present a male with a clinical phenotype of BCM carrying a novel deletion originated by a de novo mutation event in the *OPN1LW/OPN1MW* gene cluster. Herein, we describe the clinical findings of the patient, present the results from molecular genetic investigations of the patient and his family members, and discuss the putative underlying mechanism leading to this de novo mutation.

## Case presentation

**Fig. 1:**
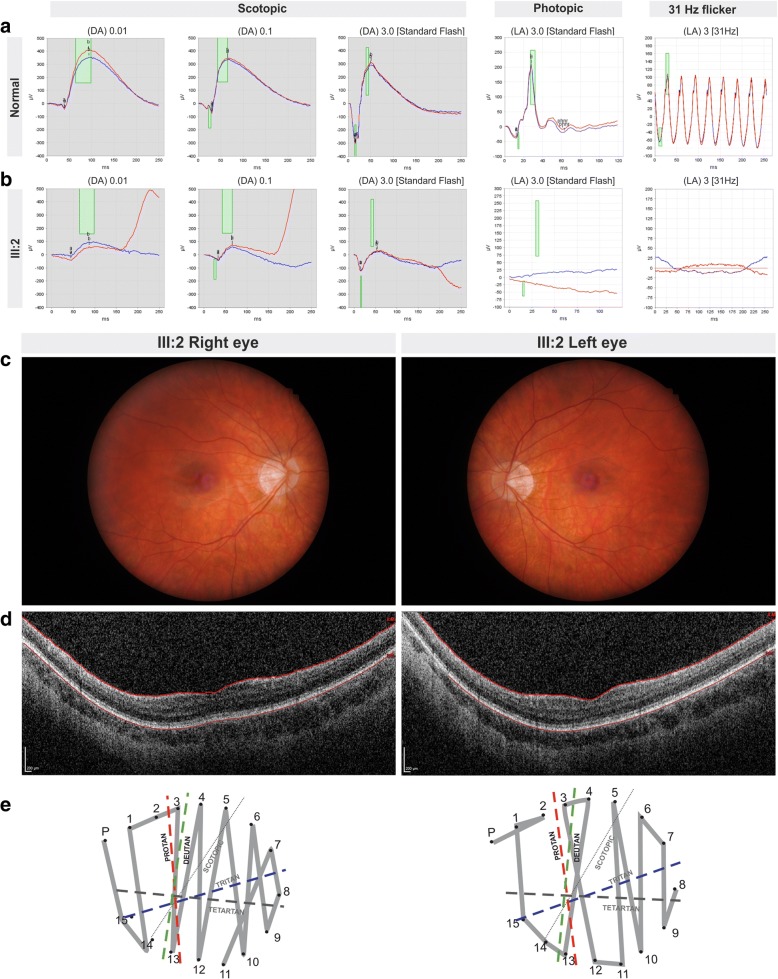
Ophthalmological findings in BCM patient III:2 **a** Full-field ERG recordings according to ISCEV Standards of a normal subject **b** ERG of the index patient (III:2) at last clinical examination (24 years old) showing mild reduction under dark-adapted (DA) scotopic conditions and undetectable responses under light-adapted (LA) photopic responses in both eyes. Strength of the light stimulus is given in photopic cd*s/m^2^, if not stated otherwise. Grey sections indicate scotopic recording conditions. Red colour represents right eye and blue the left eye. Green rectangles indicate 5–95% confidence intervals for amplitudes and implicit times of a normal population. **c** Right and left eye fundus image of the patient showing tilted optic disc. **d** Right and left eye OCT shows normal retinal architecture in the BCM patient with thinned photoreceptor layers. **e** Farnsworth Panel D-15 saturated shows confusion errors along the protan-deutan axes for both eyes

A 24-year-old male presented with photophobia since birth. No family history for colour vision defects or retinal dystrophies was reported. Myopia with an refractive error of − 5.50 D (right eye) and − 6.50 D (left eye) and astigmatism were found in the patient (III:2) at the age of 8 months along with nystagmus but devoid of strabismus. Glasses were given at the age of 1 year. Difficulties distinguishing colours were noticed by his parents at the age of 3 years. Achromatopsia was the first suspected diagnosis. At the age of 4 years occlusion therapy alternating in both eyes for 2 months was attempted to treat amblyopia but was unsuccessfull. A best-corrected visual acuity of 20/200 was measured with Snellen charts at the age of 6 years. No brain injuries were detected by magnetic resonance imaging. Visual evoked potential flash and B-scan ultrasonography performed normal for both eyes. At the age of 11 years a visual acuity of 20/250 was measured. At the latest exam at the age of 24 years, full-field light- and dark-adapted electroretinogram (ERG) recordings were performed according to the International Society for Clinical Electrophysiology of Vision (ISCEV) standard protocol [8]. Subnormal amplitudes under scotopic conditions and extinct responses under photopic conditions were observed in both eyes of the patient (III:2) in comparison to normal controls (Fig. 1a-b). A visual acuity of 20/400 was measured for both eyes with a myopic correction of − 12.00 D (right eye) and − 11.50 D (left eye). Anterior segment, pupillary reflexes and intraocular pressure revealed no abnormalities. Eye fundus examination revealed normal retinal vessels, optic nerve heads showing tilted optic discs with myopic conus and the maculae had elapsed reflex without waxy reflex (Fig. 1c). Spectral domain optical coherence tomography (SD-OCT) performed with Spectralis OCT (Heidelberg Engineering) showed normal retinal architecture with thinned photoreceptor layer (Fig. 1d). Colour vision test evaluated with the Farnsworth D-15 Colour Test revealed protan-deutan confusion errors (Fig. 1e).

**Fig. 2:**
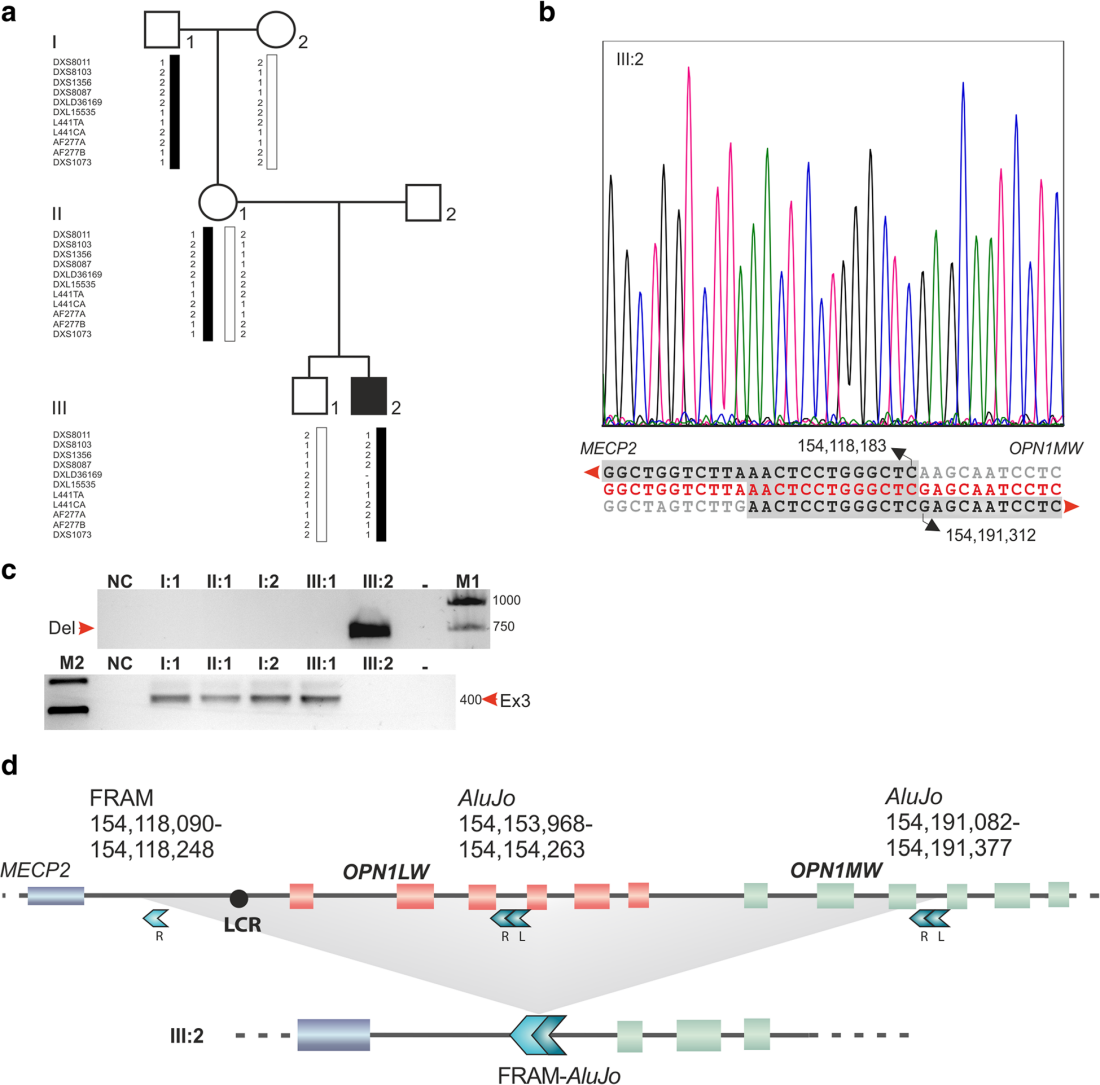
Identification of a *de novo* 73,128 bp deletion at the cone opsin gene cluster **a** Pedigree of the family with a single affected male (III:2). Reconstructed haplotypes based on microsatellite markers flanking the *OPN1LW/MW* gene cluster within Xq28 revealed transmission of the X-chromosome from the maternal grandfather (I:1) to the index patient (III:2). **b** Sanger sequencing traces of the deletion breakpoints observed in the index patient (III:2) and its sequence depicted in red letters in the alignment underneath. Wildtype sequence at the centromeric and the telomeric breakpoint are depicted in black letters at the top and the bottom of the alignment, respectively. The breakpoints define a 73,128 bp deletion (NC_000023.11:g.154,118,184_154,191,311del) including the LCR, the entire *OPN1LW* gene and the first three exons of the *OPN1MW* gene. **c** Segregation analysis demonstrated the *de novo* occurence of the deletion. A breakpoint PCR amplicon yielded only a product for patient III:2 (“Del”, upper agarose gel panel), whereas a PCR amplicon for exon 3 of *OPN1LW* and *OPN1MW* (“Ex3”, lower agarose gel panel) yielded products for the mother (II:1) and the grandfatehr (I:1) but not for the patient (III:2). M1 & M2: DNA size standards; NC: no DNA template negative control; −: empty well. **d** Schematic representation of the 73,128 bp deletion encompassing the LCR, the entire *OPN1LW* and 5′ parts of *OPN1MW* gene in the BCM patient (III:2). Red and green boxes depict individual exons of the *OPN1LW* and *OPN1MW* genes, respectively. The localization and composition of Alu elements most likely involved in the mutation event are shown as arrowheads. Physical coordinates refer to Genbank entry NC_000023.11

Mutation screening of *OPN1LW/OPN1MW* gene cluster in the patient (III:2) was performed as previously described [9]. Genotyping PCRs with genomic DNA from the patient (III:2, see Fig. 2a for a pedigree of the genetically investigated family members) revealed absence of the LCR and promoter regions of both *OPN1LW* and *OPN1MW* genes – indicative for a large deletion – but presence of the 3′ parts of the *OPN1MW* gene, namely exons 4, 5 and 6. Upon fine sequence tag site content mapping, the deletion was finally bridged with a PCR amplicon performed with primers BCM#27_F (5′- TCGACCCAGAATTAACCTCTCT -3′) and BCM#27BPR (5’-TCTAAAAATGGACAAGGATTAACCA -3′) which was sequenced with Sanger to determine the exact breakpoints in patient III:2 (Fig. 2b and Fig. 2c). The deletion, NC_000023.11:g.154,118,184_154,191,311del, encompasses 73,128 bp with the centromeric breakpoint located in the intergenic region between *MECP2* and *OPN1LW* and the telomeric breakpoint within intron 3 of *OPN1MW* (Fig. 2d).

A sequence alignment of the breakpoint junction sequence in the patient (III:2) with the corresponding non-mutant sequence sections from his grandfather (I:1) revealed an overlapping stretch of 13 bp between the centromeric and the telomeric breakpoint sequences (Fig. 2b) shared by two Alu elements. The sequence remnants embedded within the deletion breakpoints resembled the junction of two Alu elements; (1) a fossil right Alu momomer (FRAM) element at the centromeric breakpoint of the deletion, and (2) an *AluJo* element in intron 3 of *OPN1MW* at the telomeric breakpoint of the deletion (Fig. 2d). Microsatellite marker analysis revealed that the X-chromosome present in the patient (III:2) had been transmitted from his maternal grandfather (I:1, Fig. 2a). Segregation analysis performed by means of breakpoint PCR amplification showed that neither the patient’s grandfather (I:1) nor the mother (II:1) carry the deletion (Fig. 2c). The CARE guidelines were followed in reporting this case.

## Discussion and conclusions

In this report we describe a deletion spanning 73,128 bp within the human *OPN1LW/OPN1MW* cone opsin gene cluster in a male with clinical features fully compatible with BCM but lacking family history for this condition. The molecular findings were critical to establish the correct diagnosis for the patient. A more thorough clinical investigation at 24 years of age showed results concordant with a BCM phenotype (Fig. 1a-e). While there is evidence for a link between emmetropization and the M to L cone ratio [10], the mechanism(s) underlying myopia in BCM are still unsolved. Presumably due to the high myopia and the lack of cone contribution to the dark-adapted responses, a mild reduction of scotopic signals was observed (Fig. 1a-b). Previously, rod responses to dim stimuli were reported to be in the lower normal or subnormal range in BCM patients [4, 6].

The NC_000023.11:g.154,118,184_154,191,311del mutation in this patient has not been previously reported. The *de novo* nature of the deletion was confirmed by haplotype reconstruction and segregation analysis of all family members available. While the X-chromosome present in the patient (III:2) had been transmitted from his maternal grandfather (I:1, Fig. 2a), the NC_000023.11:g.154,118,184_154,191,311del mutation was absent in the patient’s grandfather (I:1) and mother (II:1, Fig. 2c). Therefore the mutation identified in the patient of this particular case represents a *de novo* event that occurred in the mother’s germline (II:1, Fig. 2c).

The breakpoint sequences of the deletion are localized within Alu repetitive elements. Based on the sequence composition prior to deletion and the actual Alu sequence remnants found at the deletion breakpoints, it is tempting to propose a mechanism underlying this *de novo* mutation event. We hypothesize that the monomeric FRAM element underwent pairing with the left arm of the bipartite *AluJo* element and induced subsequent recombination within the 13 bp of microhomology. FRAM and *AluJo* most likely promoted the origin of the deletion during meiosis through misalignment of the homologous stretch [11]. The FRAM and *AluJo* elements may have acted in this very event most likely as nucleation spots allowing mispairing and making the sequence prone to homologous recombination [11]. The resulting deletion gave rise to a hybrid FRAM-*AluJo* element. Notably, more than 95% of the Alu-mediated deletion events identified in the human genome occurred as Alu-mediated unequal homologous recombination [12]. According to the aforementioned model proposed, the Alu element mediated illegitimate recombination may have occurred either by mispairing of sister or non-sister chromatids (interchromosomal), or between elements on the same chromosome (intrachromosomal).

Although several studies have previously reported deletions including the LCR to be associated with a BCM phenotype [1, 3–7], this is the first *de novo* deletion reported to underlie a BCM phenotype. We have previously described a *de novo* intrachromosomal gene conversion event in the male germline transferring a pathogenic haplotype from *OPN1MW* to *OPN1LW* [12]. Notwithstanding, the BCM patient’s grandfather presented with a linked colour vision deficiency due to a pathogenic haplotype in *OPN1MW* and the patient’s mother was a carrier of the converted *OPN1LW* gene [13]. Our case report herein describes a BCM patient bearing a unique mutation that stem from an independent *de novo* event in a family with no history of BCM or colour vision abnormalities.

In summary, we were able to elucidate the origin of a newly emerged mutation in an isolated case of BCM. These findings emphasize the need to test for X-linked BCM in cases with a clinical diagnosis of cone dysfunction, even in the absence of a family history in order to obtain a correct clinical diagnosis and a valid basis for proper genetic counselling. The novel 73,128 bp deletion within the *OPN1LW/MW* gene cluster occurred most likely as a result of an Alu recombination-mediated mechanism. This is the first report of a *de novo* deletion resulting in BCM.

## Abbreviations

BCM: Blue Cone Monochromacy
ERG: Electroretinogram
FRAM: Fossil right Alu momomer
LCR: Locus control region
SD-OCT: Spectral domain optical coherence tomography
